# Update of the fractions of cardiovascular diseases and mental disorders attributable to psychosocial work factors in Europe

**DOI:** 10.1007/s00420-021-01737-4

**Published:** 2021-06-28

**Authors:** Isabelle Niedhammer, Hélène Sultan-Taïeb, Agnès Parent-Thirion, Jean-François Chastang

**Affiliations:** 1 INSERM, Univ Angers, Univ Rennes, EHESP, Irset (Institut de Recherche en Santé, Environnement Et Travail), UMR_S 1085, Epidemiology in Occupational Health and Ergonomics (ESTER) Team, Angers, France; 2 Human Resources Department, School of Management, Université du Québec À Montréal (UQAM), Montréal, QC Canada; 3 European Foundation for the Improvement of Living and Working Conditions (EUROFOUND), Dublin, Ireland

**Keywords:** Attributable fraction, Exposure prevalence, Job stress, Psychosocial work factors, Cardiovascular diseases, Mental disorders, Depression, Europe

## Abstract

**Objectives:**

The objectives of this study were to provide the fractions of cardiovascular diseases and mental disorders attributable to five psychosocial work exposures, i.e. job strain, effort-reward imbalance, job insecurity, long working hours, and bullying in Europe (35 countries, including 28 European Union countries), for each one and all countries together, in 2015.

**Methods:**

The prevalences of exposure were estimated using the sample of 35,571 employees from the 2015 European Working Conditions Survey (EWCS) for all countries together and each country separately. Relative risks (RR) were obtained via literature reviews and meta-analyses already published. The studied outcomes were: coronary/ischemic heart diseases (CHD), stroke, atrial fibrillation, peripheral artery disease, venous thromboembolism, and depression. Attributable fractions (AF) for each exposure and overall AFs for all exposures together were calculated.

**Results:**

The AFs of depression were all significant: job strain (17%), job insecurity (9%), bullying (7%), and effort-reward imbalance (6%). Most of the AFs of cardiovascular diseases were significant and lower than 11%. Differences in AFs were observed between countries for depression and for long working hours. Differences between genders were found for long working hours, with higher AFs observed among men than among women for all outcomes. Overall AFs taking all exposures into account ranged between 17 and 35% for depression and between 5 and 11% for CHD.

**Conclusion:**

The overall burden of depression and cardiovascular diseases attributable to psychosocial work exposures was noticeable. As these exposures are modifiable, preventive policies may be useful to reduce the burden of disease associated with the psychosocial work environment.

**Supplementary Information:**

The online version contains supplementary material available at 10.1007/s00420-021-01737-4.

## Introduction

Psychosocial work factors constitute major occupational hazards in the working populations of developed countries. They have been found to be associated with various health outcomes, including cardiovascular diseases and mental disorders, for which a high level of evidence has been provided by the literature (Kivimaki 2012; Madsen 2017). However, the burden of diseases attributable to these factors remains understudied for Europe and European countries. The estimation of such a burden may be useful for at least two reasons. First, this estimation may inform and guide stakeholders to take decisions on preventive measures (Niedhammer et al. 2014b). Second, this estimation may be a preliminary step for the calculation of the costs of diseases attributable to such factors (Sultan-Taïeb et al. 2013; Sultan-Taïeb and Niedhammer 2013). Nevertheless, the evaluation of such a burden, through the calculation of attributable fractions, implies causality between exposure and outcome, which is strongly dependent on the level of evidence available.

Some years ago, we published a paper presenting first estimates of the fractions of cardiovascular diseases and mental disorders attributable to psychosocial work exposures for Europe as a whole and each country separately (Niedhammer et al. 2014a). The studied exposures were job strain, effort-reward imbalance (ERI), and job insecurity. Indeed, these exposures represent concepts that are both well-known and widely used. Job strain is probably the most prominent psychosocial work exposure, derived from the job strain model by Karasek (Karasek et al. 1998) and defined by the combination of high psychological demands and low decision latitude. ERI from the ERI model (Siegrist 2004) is the exposure defined by an excess of effort made compared to low levels of reward obtained. Job insecurity is generally defined by the fear or threat of job loss (Bartley and Ferrie 2001). Since 2014, other psychosocial work factors have emerged and the literature has grown, making the study of other exposures possible. At least two major exposures have been studied within the past years, which are long working hours and workplace bullying. Long working hours are defined by an excess of working hours, using thresholds that may vary according to studies (van der Hulst 2003). Workplace bullying is characterized by various aspects of psychological and physical violence at the workplace (Einarsen 2000).

In our earlier publication (Niedhammer et al. 2014a), we found that the highest significant attributable fraction was the fraction of mental disorders attributable to job strain in Europe (18%). The fractions of these disorders attributable to ERI (9%) and job insecurity (5%) were also significantly different from zero. The fraction of cardiovascular diseases attributable to job strain was 4% and significant as well. Almost no difference between European countries was observed in these fractions.

The present study added to the previous one by providing up-to-date estimates for exposure prevalences and attributable fractions with the use of more recent data (2015 instead of 2005 previously), covering Europe and all European countries more widely (35 European countries instead of 31 previously), and enlarging the study of psychosocial work exposures to the more recent concepts of long working hours and workplace bullying.

The objectives of this study were thus to provide up-to-date estimates of the fractions of cardiovascular diseases and mental disorders attributable to the five aforementioned psychosocial work exposures in Europe as a whole and in 35 European countries separately.

## Methods

### Prevalence of exposure

The study used the data from the 2015 European Working Conditions survey (EWCS) to assess the prevalence of exposure (Pe) to psychosocial work factors. This survey is a periodical survey on working conditions among the European working populations set up by the European Foundation for the improvement of living and working conditions (EUROFOUND) since 1990. This survey is an important source of information for the assessment of occupational exposures in European countries, as it relies on the same protocol and questionnaire all over Europe. The sixth edition of the survey was performed in 2015 and covered 35 European countries, including the 28 European Union (EU) countries (the UK was still part of the EU in 2015). The sample was representative of all workers, employees and self-employed workers, aged 15 or more, living in private households, and in employment at the time of the survey (i.e. who worked during the week preceding the interview according to Eurostat definition). The sampling procedure was a multistage stratified random sampling. More information about the survey protocol and sampling design can be found elsewhere (EUROFOUND 2017). The survey sample included 43,850 workers. The sample was restricted to employees, as self-employed workers were not always asked the same questions in the survey. Consequently, the study sample included 35,571 employees, including 17,109 men and 18,453 women. There were 9 employees without information about gender, and their data were used when all men and women together were studied. The sample size of employees according to country ranged between 553 (Albania) and 2762 (Spain) with a mean value of 1016 in each country (Supplementary Table S1).

Five psychosocial work factors were assessed using the 2015 EWCS data as follows: job strain, ERI, job insecurity, long working hours, and bullying. The list of the items from the 2015 questionnaire used to construct these factors is presented in Appendix. The construction of the factors was close but not strictly identical to the construction of the factors of job strain, ERI, and job insecurity in our previous publication (Niedhammer et al. 2014a), as some items were removed or added in the 2015 questionnaire compared to the 2005 questionnaire. To make the factor construction possible, the coding of some items (with reverse formulation) was reversed and the coding was made homogeneous if the coding was different between items. Job strain was defined by the combination of high psychological demands and low decision latitude following the job strain model (Karasek et al. 1998). Psychological demands were based on a sum score of five items (Cronbach’s alpha coefficient: 0.64). Decision latitude was constructed using a weighted sum of skill discretion (three items) and decision authority (eight items), the two components being given the same weight (Cronbach’s alpha coefficient: 0.78). Exposure to high demands and low latitude was defined using the median of the distribution among the total sample. ERI was defined by a weighted ratio of effort and reward over 1, i.e. an imbalance between high effort and low reward (Siegrist et al. 2004). Effort was measured using six items (Cronbach’s alpha coefficient: 0.56). Reward was measured by a weighted sum giving the same weight to the following three components: reward (five items), job promotion (three items), and job security (one item) (Cronbach’s alpha coefficient: 0.73). There was no major change in Cronbach’s alpha coefficients between countries. Job insecurity was measured using the same one item (strongly or tend to agree to lose job in the next 6 months). Long working hours (one item) was defined by 55 h or more a week, this threshold being chosen to be consistent with the literature. Bullying was measured using one item, which was exposure to workplace bullying/harassment within the past 12 months.

The prevalence of exposure to these five factors was calculated from weighted data to provide estimates that were representative for Europe as a whole (35 countries), for the 28 EU countries, and for each country. The calculation of 95% confidence intervals also took weighting into account. The difference in exposure between countries was tested using the Wald test. The main analyses were based on the total sample of men and women. Differences in the prevalence of exposure between genders were tested using the Rao–Scott Chi-Square test.

### Relative risk

Relative risk (RR) estimates were obtained from the most recent literature reviews or if not available from studies of the IPD-Work consortium (Kivimaki et al. 2015b) that provided pooled RR estimates from various cohort studies from a range of countries. Fifteen literature reviews and IPD-Work consortium studies were used to provide RRs for a number of associations between psychosocial work factors and health outcomes related to cardiovascular diseases and mental disorders. These pooled RRs were based on prospective studies and were related to health outcomes that were clearly defined, i.e. that could be found in the International Classification of Diseases (ICD-10). The studied exposures were the five exposures measured using the 2015 EWCS data. The pooled RRs used in the present study were those that were adjusted for gender, age, and socioeconomic status (SES), or the closest adjustment. If various adjusted pooled RRs were available for a given exposure-outcome association in the literature, we retained the one that was adjusted for gender, age, and SES. Careful consideration was given to gender-stratified results or to all information provided by the authors on gender differences in RRs. However, the gender differences turned out to be either unexplored in the selected literature or non-significant i.e. the gender-related interactions were found to be non-significant (meaning no difference in RRs between men and women). Consequently, the RRs and their 95% confidence intervals for men and women together were used and are presented in Table 1. The outcomes related to cardiovascular diseases were coronary/ischemic heart disease (CHD), stroke, atrial fibrillation, peripheral artery disease, and venous thromboembolism, and the outcome for mental disorders was depression. All RRs were significant except for the two associations of job strain-overall stroke and job strain–hemorrhage stroke.

**Table 1:** Summary adjusted relative risks and 95% confidence intervals

	RR	95% CI	
**CHD**			
Job strain (Kivimaki et al. 2012)^$^	1.17	1.05	1.31
Effort-reward imbalance (Dragano 2017)^$^	1.19	1.04	1.38
Job insecurity (Virtanen et al. 2013)^1$^	1.32	1.09	1.59
Long working hours (Kivimaki 2015a; Li et al. 2020)^2$^	1.13	1.02	1.26
**Stroke**			
Job strain (Fransson 2015)			
Overall stroke^3^	1.09	0.94	1.26
Ischemic stroke	1.18	1.00	1.39
Hemorrhagic stroke	0.95	0.72	1.27
Long working hours (Descatha 2020)^2$^			
Overall stroke	1.35	1.13	1.61
**Atrial fibrillation**			
Long working hours (Kivimaki 2017)^$^	1.42	1.13	1.80
**Peripheral artery disease**			
Job strain (Heikkila et al. 2020)^3$^	1.46	1.17	1.83
**Venous thromboembolism**			
Long working hours (Kivimaki et al. 2018)^4^	1.5	1.1	2.1
**Depression**			
Job strain (Madsen et al. 2017)^5^	1.77	1.47	2.13
Effort-reward imbalance (Rugulies et al. 2017)^6^	1.68	1.40	2.01
Job insecurity (Ronnblad et al. 2019)	1.61	1.29	2.00
Long working hours (Virtanen et al. 2018)^7$^	1.14	1.03	1.25
Bullying (Theorell et al. 2015)^8^	2.82	2.21	3.59

Adjustment for gender, age, and SES, except:

^1^Adjusted for age

^2^Adjusted for at least gender, age, and SES

^3^Adjusted for gender and age

^4^Adjusted for gender, age, cohort, and SES

^5^Adjusted variables varied by study (published studies on clinical depression only)

^6^From least adjusted study-specific estimates

^7^Adjusted for gender, age, SES, and marital status or the closest

^8^From the least adjusted model from each study

^$^No significant gender differences in the RRs

### Attributable fractions

Attributable fraction (AF) provides an estimate of “the fraction of disease cases that is attributable to an exposure in a population and that would not have been observed if the exposure had been non-existent” (Nurminen and Karjalainen 2001). The calculation of AF implies that there is a causal association between exposure and outcome, but appears justified if there is a high level of evidence, though non-causal. AF for each exposure-outcome pair was calculated using the following formula (Nurminen and Karjalainen 2001), with Pe prevalence of exposure and RR relative risk:

1 $$AF = Pe\left( {RR - 1} \right)/\left[ {1 + Pe\left( {RR - 1} \right)} \right]$$

The AFs were calculated for Europe as a whole (all 35 countries), for the 28 EU countries, and for each country. 95% confidence intervals were calculated using simulation-modelling techniques that were presented in our previous study (Niedhammer et al. 2014a). Briefly, we simulated Pe and log(RR) through a normal distribution in order to provide the mean and variance and consequently the 95% confidence interval of the AFs. The difference in AFs between countries was tested using the Wald test. As the RRs for a given exposure-outcome association were assumed to be the same for all countries, the differences in AFs between countries were related to the differences in exposure prevalence between countries. Similarly, as the RRs were assumed to be the same for both genders, the differences in AFs between genders were related to the differences in exposure prevalence between men and women. Gender-stratified AFs were calculated only if the difference in exposure prevalence was significant between genders. The difference in AFs between men and women was tested using the Wald test.

The direct calculation of the overall fraction of a given disease attributable to all studied psychosocial work exposures together was not possible as no data was available for the RRs associated with combined exposures. This is why we used two estimates that can be considered as minimum and maximum values for the overall fraction. Indeed, employees may be exposed to more than one psychosocial work factor, or in other words, there may be overlap/correlation between exposures. The minimum value was obtained from the highest individual AF observed for each exposure studied alone (the underlying assumption was the total dependence/overlap between all exposures). The maximum value was calculated from Miettinen’s formula (Miettinen 1974), that ensures that the overall AF does not exceed 100% (contrary to the mere sum of the individual AFs for each exposure) and assumes the independence (i.e. no overlap) between *p* exposures:

2 $$Overall~AF~\left( {max} \right) = 1 - \mathop \prod \limits_{{i = 1}}^{p} \left[ {1 - AF\left( i \right)} \right]$$

All statistical analyses were performed using SAS software.

## Results

### Results for job strain (Table 2)

**Table 2:** Fractions of cardiovascular diseases and mental disorders attributable to job strain in Europe

%	Exposure prevalence		CHD		Overall stroke		Peripheral artery disease		Depression	
	Pe	95% CI	AF	95% CI	AF	95% CI	AF	95% CI	AF	95% CI
Albania	39.43	[34.68–44.18]	**6.38**	**[1.84–10.93]**	3.38	[−2.52–9.27]	**15.44**	**[6.10–24.78]**	**23.25**	**[15.35–31.14]**
Austria	22.45	[19.23–25.68]	**3.75**	**[1.00–6.51]**	1.97	[−1.49–5.44]	**9.48**	**[3.33–15.64]**	**14.76**	**[9.12–20.41]**
Belgium	20.16	[18.26–22.07]	**3.38**	**[0.92–5.85]**	1.78	[−1.34–4.90]	**8.61**	**[3.04–14.19]**	**13.47**	**[8.39–18.55]**
Bulgaria	21.30	[18.27–24.33]	**3.57**	**[0.94–6.19]**	1.88	[−1.42–5.17]	**9.05**	**[3.15–14.95]**	**14.12**	**[8.68–19.55]**
Croatia	35.20	[31.38–39.01]	**5.74**	**[1.64–9.85]**	3.03	[−2.27–8.33]	**14.04**	**[5.44–22.64]**	**21.30**	**[13.94–28.66]**
Cyprus	43.15	[39.28–47.02]	**6.94**	**[2.05–11.83]**	3.68	[−2.73–10.08]	**16.63**	**[6.76–26.50]**	**24.89**	**[16.75–33.03]**
Czech Rep	26.79	[23.29–30.29]	**4.44**	**[1.21–7.67]**	2.34	[−1.76–6.44]	**11.09**	**[4.04–18.14]**	**17.11**	**[10.80–23.42]**
Denmark	19.19	[16.41–21.97]	**3.23**	**[0.84–5.61]**	1.70	[−1.29–4.68]	**8.23**	**[2.82–13.65]**	**12.91**	**[7.86–17.95]**
Estonia	21.28	[18.00–24.57]	**3.56**	**[0.93–6.20]**	1.88	[−1.42–5.17]	**9.04**	**[3.13–14.95]**	**14.11**	**[8.63–19.58]**
Finland	16.31	[13.56–19.06]	**2.76**	**[0.70–4.82]**	1.45	[−1.11–4.00]	**7.09**	**[2.33–11.85]**	**11.19**	**[6.65–15.73]**
France	24.33	[21.82–26.83]	**4.05**	**[1.11–6.99]**	2.13	[−1.60–5.87]	**10.19**	**[3.69–16.69]**	**15.80**	**[9.98–21.62]**
FYROM	29.44	[25.65–33.23]	**4.86**	**[1.34–8.37]**	2.56	[−1.92–7.05]	**12.04**	**[4.47–19.62]**	**18.48**	**[11.79–25.17]**
Germany	22.01	[19.81–24.22]	**3.68**	**[1.00–6.36]**	1.94	[−1.46–5.33]	**9.32**	**[3.32–15.32]**	**14.52**	**[9.10–19.95]**
Greece	46.95	[42.51–51.39]	**7.50**	**[2.24–12.76]**	3.98	[−2.95–10.90]	**17.81**	**[7.37–28.25]**	**26.48**	**[17.98–34.98]**
Hungary	31.44	[27.77–35.12]	**5.17**	**[1.45–8.89]**	2.73	[−2.04–7.50]	**12.75**	**[4.81–20.69]**	**19.49**	**[12.57–26.41]**
Ireland	25.92	[22.30–29.55]	**4.30**	**[1.16–7.45]**	2.27	[−1.71–6.24]	**10.78**	**[3.89–17.67]**	**16.65**	**[10.44–22.86]**
Italy	22.78	[19.79–25.77]	**3.80**	**[1.02–6.59]**	2.00	[−1.51–5.52]	**9.61**	**[3.40–15.82]**	**14.95**	**[9.29–20.60]**
Latvia	15.16	[12.45–17.88]	**2.57**	**[0.64–4.50]**	1.35	[−1.03–3.73]	**6.63**	**[2.14–11.12]**	**10.49**	**[6.16–14.82]**
Lithuania	27.56	[24.01–31.10]	**4.56**	**[1.25–7.87]**	2.40	[−1.81–6.62]	**11.37**	**[4.17–18.57]**	**17.51**	**[11.10–23.93]**
Luxembourg	21.23	[18.21–24.25]	**3.56**	**[0.94–6.17]**	1.87	[−1.42–5.16]	**9.02**	**[3.14–14.90]**	**14.08**	**[8.66–19.50]**
Malta	15.46	[12.67–18.26]	**2.62**	**[0.65–4.59]**	1.38	[−1.05–3.80]	**6.75**	**[2.19–11.32]**	**10.68**	**[6.27–15.08]**
Montenegro	33.29	[29.04–37.54]	**5.45**	**[1.53–9.38]**	2.88	[−2.16–7.91]	**13.39**	**[5.09–21.68]**	**20.39**	**[13.18–27.60]**
Netherlands	15.29	[12.42–18.16]	**2.59**	**[0.64–4.54]**	1.36	[−1.04–3.76]	**6.68**	**[2.15–11.22]**	**10.57**	**[6.18–14.96]**
Norway	13.12	[10.91–15.34]	**2.23**	**[0.56–3.91]**	1.17	[−0.90–3.24]	**5.80**	**[1.85–9.74]**	**9.22**	**[5.39–13.04]**
Poland	24.36	[21.43–27.29]	**4.06**	**[1.10–7.01]**	2.14	[−1.61–5.88]	**10.20**	**[3.67–16.73]**	**15.82**	**[9.93–21.70]**
Portugal	26.16	[22.37–29.95]	**4.34**	**[1.17–7.51]**	2.29	[−1.73–6.30]	**10.86**	**[3.91–17.81]**	**16.78**	**[10.51–23.05]**
Romania	36.83	[32.76–40.90]	**5.99**	**[1.72–10.27]**	3.17	[−2.36–8.70]	**14.58**	**[5.70–23.47]**	**22.06**	**[14.50–29.63]**
Serbia	28.29	[24.17–32.41]	**4.68**	**[1.27–8.08]**	2.47	[−1.86–6.79]	**11.63**	**[4.25–19.01]**	**17.89**	**[11.29–24.49]**
Slovakia	29.40	[25.58–33.21]	**4.85**	**[1.34–8.36]**	2.56	[−1.92–7.04]	**12.03**	**[4.46–19.60]**	**18.46**	**[11.77–25.15]**
Slovenia	24.23	[21.54–26.92]	**4.04**	**[1.10–6.97]**	2.13	[−1.60–5.85]	**10.15**	**[3.66–16.64]**	**15.75**	**[9.92–21.57]**
Spain	33.68	[31.57–35.80]	**5.51**	**[1.59–9.43]**	2.91	[−2.17–7.99]	**13.52**	**[5.25–21.80]**	**20.59**	**[13.55–27.62]**
Sweden	16.07	[13.46–18.68]	**2.72**	**[0.69–4.75]**	1.43	[−1.09–3.94]	**7.00**	**[2.31–11.69]**	**11.05**	**[6.58–15.52]**
Switzerland	21.44	[18.45–24.43]	**3.59**	**[0.95–6.23]**	1.89	[−1.43–5.20]	**9.10**	**[3.18–15.02]**	**14.19**	**[8.75–19.64]**
Turkey	35.96	[32.93–38.99]	**5.86**	**[1.69–10.03]**	3.10	[−2.30–8.50]	**14.30**	**[5.60–22.99]**	**21.67**	**[14.30–29.03]**
UK	27.26	[24.47–30.05]	**4.51**	**[1.25–7.78]**	2.38	[−1.78–6.54]	**11.26**	**[4.16–18.37]**	**17.36**	**[11.08–23.64]**
Total (35 countries)	25.92	[25.16–26.68]	**4.30**	**[1.21–7.39]**	2.27	[−1.69–6.23]	**10.78**	**[4.01–17.55]**	**16.66**	**[10.73–22.58]**
p-value^3^	***		ns		ns		ns		***	
Total (28 EU countries)	25.16	[24.35–25.97]	**4.18**	**[1.18–7.19]**	2.20	[-1.64–6.05]	**10.50**	**[3.88–17.11]**	**16.25**	**[10.44–22.06]**
p-value^3^	***		ns		ns		ns		**	

Bold: AF significantly different from 0 at 5%. *p* value for the comparison between countries: *p < 0.05; **p < 0.01; *** *p* < 0.001; *ns* non-significant

*FYROM* Former Yugoslav Republic of Macedonia, *Pe* prevalence of exposure, *AF* attributable fraction

The overall prevalence of job strain was 26% in Europe (35 countries) and there was a significant difference between countries. The lowest prevalence of exposure (less than 17%) was observed in the Scandinavian countries, Latvia, Malta, and the Netherlands, and the highest (more than 40%) in Cyprus and Greece. The fraction of CHD attributable to job strain was 4% and significantly different from zero. There was no difference in this AF between countries. The AF for overall stroke was not significantly different from zero and there was no difference between countries. Supplementary results for ischemic and hemorrhagic stroke (Supplementary Table S2) showed that the AFs were significant neither for ischemic stroke nor for hemorrhagic stroke. The AF for peripheral artery disease was significant (11%), and there was no difference between countries. The AF for depression was 17% and was significant. This AF displayed significant differences between countries.

### Results for ERI (Table 3)

**Table 3:** Fractions of cardiovascular diseases and mental disorders attributable to effort-reward imbalance (ERI) in Europe

%	Exposure prevalence		CHD		Depression	
	Pe	95% CI	AF	95% CI	AF	95% CI
Albania	13.36	[10.10–16.63]	**2.60**	**[0.35–4.86]**	**8.34**	**[4.45–12.23]**
Austria	8.79	[6.69–10.89]	**1.73**	**[0.22–3.24]**	**5.66**	**[2.95–8.36]**
Belgium	7.90	[6.60–9.19]	**1.56**	**[0.23–2.89]**	**5.11**	**[2.80–7.42]**
Bulgaria	5.47	[3.69–7.24]	**1.08**	**[0.10–2.07]**	**3.60**	**[1.68–5.52]**
Croatia	13.24	[10.44–16.03]	**2.58**	**[0.37–4.79]**	**8.27**	**[4.52–12.02]**
Cyprus	11.97	[9.50–14.44]	**2.34**	**[0.34–4.34]**	**7.54**	**[4.11–10.97]**
Czech Rep	7.00	[4.87–9.13]	**1.38**	**[0.14–2.62]**	**4.56**	**[2.20–6.92]**
Denmark	6.07	[4.48–7.66]	**1.20**	**[0.14–2.26]**	**3.98**	**[2.00–5.96]**
Estonia	6.91	[4.88–8.95]	**1.37**	**[0.15–2.59]**	**4.51**	**[2.20–6.81]**
Finland	6.71	[4.86–8.56]	**1.33**	**[0.15–2.50]**	**4.38**	**[2.18–6.58]**
France	11.45	[9.62–13.27]	**2.24**	**[0.34–4.14]**	**7.24**	**[4.06–10.42]**
FYROM	8.85	[6.67–11.04]	**1.74**	**[0.22–3.26]**	**5.70**	**[2.95–8.44]**
Germany	6.80	[5.53–8.08]	**1.35**	**[0.19–2.50]**	**4.44**	**[2.38–6.50]**
Greece	14.23	[11.28–17.17]	**2.77**	**[0.41–5.13]**	**8.83**	**[4.87–12.79]**
Hungary	9.54	[7.10–11.98]	**1.88**	**[0.23–3.52]**	**6.11**	**[3.15–9.07]**
Ireland	11.59	[8.81–14.37]	**2.27**	**[0.30–4.23]**	**7.32**	**[3.88–10.76]**
Italy	10.37	[8.26–12.48]	**2.03**	**[0.29–3.78]**	**6.60**	**[3.58–9.63]**
Latvia	5.64	[4.03–7.26]	**1.12**	**[0.12–2.12]**	**3.71**	**[1.81–5.61]**
Lithuania	5.52	[3.81–7.23]	**1.10**	**[0.11–2.08]**	**3.64**	**[1.73–5.55]**
Luxembourg	7.83	[5.83–9.82]	**1.54**	**[0.19–2.90]**	**5.07**	**[2.59–7.55]**
Malta	7.93	[5.91–9.95]	**1.56**	**[0.19–2.94]**	**5.14**	**[2.63–7.64]**
Montenegro	14.89	[11.68–18.10]	**2.89**	**[0.42–5.36]**	**9.21**	**[5.06–13.35]**
Netherlands	12.08	[9.45–14.71]	**2.36**	**[0.33–4.39]**	**7.61**	**[4.12–11.10]**
Norway	3.72	[2.46–4.99]	**0.74**	**[0.06–1.42]**	**2.48**	**[1.12–3.85]**
Poland	9.17	[7.15–11.20]	**1.80**	**[0.24–3.36]**	**5.89**	**[3.13–8.65]**
Portugal	8.11	[5.47–10.75]	**1.60**	**[0.16–3.04]**	**5.24**	**[2.49–8.00]**
Romania	7.44	[4.96–9.91]	**1.47**	**[0.14–2.80]**	**4.83**	**[2.26–7.40]**
Serbia	14.79	[11.69–17.90]	**2.87**	**[0.42–5.32]**	**9.15**	**[5.05–13.25]**
Slovakia	7.85	[5.64–10.07]	**1.55**	**[0.18–2.92]**	**5.09**	**[2.53–7.64]**
Slovenia	14.85	[12.66–17.03]	**2.88**	**[0.46–5.30]**	**9.18**	**[5.26–13.10]**
Spain	13.86	[12.30–15.42]	**2.70**	**[0.44–4.95]**	**8.63**	**[5.00–12.25]**
Sweden	10.02	[7.91–12.13]	**1.97**	**[0.27–3.66]**	**6.40**	**[3.44–9.36]**
Switzerland	8.29	[6.24–10.35]	**1.63**	**[0.21–3.06]**	**5.36**	**[2.76–7.95]**
Turkey	15.60	[13.27–17.92]	**3.02**	**[0.49–5.56]**	**9.60**	**[5.52–13.68]**
UK	11.27	[9.37–13.18]	**2.21**	**[0.33–4.08]**	**7.14**	**[3.97–10.30]**
Total (35 countries)	9.83	[9.47–10.18]	**1.93**	**[0.32–3.54]**	**6.28**	**[3.64–8.92]**
p-value	***		ns		***	
Total (28 EU countries)	9.70	[9.16–10.25]	**1.91**	**[0.31–3.50]**	**6.21**	**[3.59–8.83]**
p-value	***		ns		**	

Bold: AF significantly different from 0 at 5%. *p* value for the comparison between countries: *p < 0.05; **p < 0.01; *** *p* < 0.001; *ns* non-significant

*FYROM* Former Yugoslav Republic of Macedonia, *Pe* prevalence of exposure, *AF* attributable fraction

The prevalence of ERI was 10% in Europe (35 countries) and there were differences in this prevalence between countries. The lowest prevalence of exposure was found in Norway (4%) and the highest in Turkey (16%). The significant fraction of CHD attributable to ERI was 2% and this fraction did not differ between countries. The AF for depression was 6% and was significantly different from zero. This AF was different between countries.

### Results for job insecurity (Table 4)

**Table 4:** Fractions of cardiovascular diseases and mental disorders attributable to job insecurity in Europe

%	Exposure prevalence		CHD		Depression	
	Pe	95% CI	AF	95% CI	AF	95% CI
Albania	21.29	[17.32–25.26]	**6.36**	**[1.57–11.15]**	**11.49**	**[5.31–17.66]**
Austria	10.47	[8.10–12.83]	**3.25**	**[0.69–5.80]**	**6.03**	**[2.51–9.54]**
Belgium	14.72	[12.96–16.47]	**4.50**	**[1.11–7.89]**	**8.26**	**[3.79–12.73]**
Bulgaria	12.10	[9.54–14.66]	**3.73**	**[0.82–6.64]**	**6.90**	**[2.94–10.85]**
Croatia	19.07	[16.09–22.05]	**5.74**	**[1.43–10.05]**	**10.42**	**[4.83–16.02]**
Cyprus	14.15	[11.39–16.91]	**4.33**	**[1.00–7.67]**	**7.96**	**[3.49–12.44]**
Czech Rep	17.50	[14.43–20.57]	**5.30**	**[1.28–9.31]**	**9.65**	**[4.38–14.92]**
Denmark	11.08	[8.91–13.24]	**3.43**	**[0.76–6.10]**	**6.35**	**[2.72–9.99]**
Estonia	18.61	[15.74–21.48]	**5.61**	**[1.40–9.83]**	**10.20**	**[4.72–**15.68]
Finland	15.47	[12.79–18.16]	**4.72**	**[1.12–8.31]**	**8.64**	**[3.88–13.41]**
France	13.26	[11.29–15.24]	**4.08**	**[0.97–7.18]**	**7.51**	**[3.36–11.65]**
FYROM	18.54	[15.31–21.76]	**5.59**	**[1.37–9.82]**	**10.16**	**[4.65–15.68]**
Germany	9.31	[7.83–10.79]	**2.90**	**[0.66–5.14]**	**5.40**	**[2.34–8.46]**
Greece	23.49	[19.66–27.31]	**6.97**	**[1.79–12.14]**	**12.51**	**[5.94–19.09]**
Hungary	15.45	[12.56–18.33]	**4.71**	**[1.11–8.31]**	**8.63**	**[3.84–13.41]**
Ireland	13.73	[10.88–16.58]	**4.21**	**[0.95–7.47]**	**7.75**	**[3.36–12.14]**
Italy	21.71	[18.68–24.75]	**6.48**	**[1.67–11.29]**	**11.69**	**[5.55–17.83]**
Latvia	21.21	[18.06–24.37]	**6.34**	**[1.62–11.06]**	**11.45**	**[5.40–17.50]**
Lithuania	14.09	[11.13–17.04]	**4.32**	**[0.98–7.65]**	**7.93**	**[3.44–12.42]**
Luxembourg	10.71	[8.14–13.29]	**3.32**	**[0.69–5.95]**	**6.16**	**[2.54–9.78]**
Malta	8.45	[6.14–10.77]	**2.64**	**[0.51–4.77]**	**4.93**	**[1.92–7.93]**
Montenegro	17.97	[14.27–21.67]	**5.43**	**[1.28–9.58]**	**9.88**	**[4.42–15.35]**
Netherlands	24.42	[20.99–27.85]	**7.22**	**[1.90–12.54]**	**12.94**	**[6.24–19.65]**
Norway	10.21	[8.22–12.20]	**3.17**	**[0.70–5.64]**	**5.89**	**[2.51–9.27]**
Poland	23.97	[21.03–26.91]	**7.10**	**[1.88–12.31]**	**12.74**	**[6.17–19.31]**
Portugal	20.16	[16.29–24.03]	**6.05**	**[1.47–10.62]**	**10.95**	**[5.01–16.88]**
Romania	15.22	[12.06–18.38]	**4.64**	**[1.06–8.22]**	**8.51**	**[3.73–13.29]**
Serbia	24.98	[21.14–28.82]	**7.37**	**[1.93–12.81]**	**13.20**	**[6.34–20.05]**
Slovakia	8.06	[5.71–10.40]	**2.52**	**[0.47–4.58]**	**4.71**	**[1.80–7.62]**
Slovenia	27.32	[24.45–30.19]	**8.00**	**[2.19–13.81]**	**14.25**	**[7.06–21.44]**
Spain	25.51	[23.50–27.53]	**7.52**	**[2.05–12.98]**	**13.44**	**[6.65–20.24]**
Sweden	14.67	[12.15–17.18]	**4.48**	**[1.06–7.91]**	**8.23**	**[3.68–12.79]**
Switzerland	12.00	[9.53–14.47]	**3.70**	**[0.82–6.58]**	**6.84**	**[2.93–10.76]**
Turkey	16.45	[14.18–18.72]	**5.00**	**[1.23–8.76]**	**9.13**	**[4.20–14.07]**
UK	12.11	[10.07–14.15]	**3.74**	**[0.86–6.61]**	**6.90**	**[3.03–10.77]**
Total (35 countries)	16.56	[16.10–17.01]	**5.03**	**[1.30–8.76]**	**9.19**	**[4.36–14.02]**
p-value	***		ns		*	
Total (28 EU countries)	15.71	[15.05–16.37]	**4.79**	**[1.23–8.35]**	**8.76**	**[4.13–13.40]**
p-value	***		ns		ns	

Bold: AF significantly different from 0 at 5%. *p* value for the comparison between countries: *p < 0.05; **p < 0.01; *** *p* < 0.001 *ns* non-significant

*FYROM* Former Yugoslav Republic of Macedonia, *Pe* prevalence of exposure, *AF* attributable fraction

The prevalence of job insecurity was 17% in Europe (35 countries), and there were differences in this prevalence between countries. The lowest values were found in Germany, Malta, and Slovakia (less than 10%) and the highest in Slovenia and Spain (more than 25%). The fraction of CHD attributable to job insecurity (5%) was significantly different from zero, and there was no difference between countries. The AF for depression was 9% and was significantly different from zero. There were differences in the AFs between the 35 European countries, but no difference between the 28 EU countries.

### Results for long working hours (Table 5)

**Table 5:** Fractions of cardiovascular diseases and mental disorders attributable to long working hours in Europe

%	Exposure prevalence		CHD		Overall stroke		Atrial fibrillation		Venous thromboembolism		Depression	
	Pe	95% CI	AF	95% CI	AF	95% CI	AF	95% CI	AF	95% CI	AF^2^	95% CI
Albania	16.51	[13.04–19.98]	**2.17**	**[0.22–4.13]**	**5.49**	**[1.79–9.19]**	**5.49**	**[1.79–9.19]**	**8.05**	**[0.97–15.12]**	**2.19**	**[0.39–3.99]**
Austria	1.51	[0.63–2.39]	0.20	[−0.02–0.43]	**0.53**	**[0.05–1.02]**	**0.53**	**[0.05–1.02]**	0.81	[−0.09–1.71]	**0.21**	**[0.00–0.42]**
Belgium	3.08	[2.25–3.91]	**0.42**	**[0.03–0.80]**	**1.08**	**[0.29–1.86]**	**1.08**	**[0.29–1.86]**	**1.63**	**[0.07–3.20]**	**0.42**	**[0.06–0.78]**
Bulgaria	6.38	[4.54–8.21]	**0.85**	**[0.06–1.65]**	**2.20**	**[0.60–3.80]**	**2.20**	**[0.60–3.80]**	**3.31**	**[0.18–6.43]**	**0.86**	**[0.12–1.60]**
Croatia	6.01	[3.85–8.16]	**0.80**	**[0.03–1.58]**	**2.08**	**[0.50–3.65]**	**2.54**	**[0.42–4.67]**	**3.12**	**[0.08–6.16]**	**0.81**	**[0.09–1.53]**
Cyprus	2.30	[1.32–3.28]	**0.31**	**[0.00–0.62]**	**0.81**	**[0.16–1.46]**	**0.99**	**[0.12–1.87]**	1.23	[−0.03–2.48]	**0.31**	**[0.02–0.60]**
Czech Rep	5.06	[3.09–7.02]	**0.68**	**[0.02–1.34]**	**1.76**	**[0.39–3.12]**	**2.15**	**[0.31–3.98]**	**2.64**	**[0.02–5.26]**	**0.68**	**[0.07–1.30]**
Denmark	2.56	[1.54–3.57]	**0.34**	**[0.01–0.68]**	**0.90**	**[0.19–1.60]**	**1.10**	**[0.15–2.06]**	1.36	[−0.01–2.73]	**0.35**	**[0.03–0.66]**
Estonia	3.36	[2.06–4.66]	**0.45**	**[0.01–0.89]**	**1.17**	**[0.26–2.09]**	**1.44**	**[0.20–2.68]**	**1.77**	**[0.00–3.55]**	**0.45**	**[0.04–0.87]**
Finland	2.44	[1.34–3.54]	**0.33**	**[0.00–0.66]**	**0.86**	**[0.15–1.56]**	**1.05**	**[0.11–1.99]**	1.30	[−0.05–2.64]	**0.33**	**[0.02–0.64]**
France	3.51	[2.36–4.65]	**0.47**	**[0.02–0.92]**	**1.22**	**[0.31–2.14]**	**1.50**	**[0.25–2.75]**	**1.85**	**[0.05–3.66]**	**0.47**	**[0.06–0.89]**
FYROM	8.27	[5.93–10.61]	**1.10**	**[0.08–2.13]**	**2.84**	**[0.80–4.87]**	**3.46**	**[0.70–6.22]**	**4.24**	**[0.27–8.20]**	**1.11**	**[0.16–2.06]**
Germany	1.23	[0.61–1.84]	0.17	[−0.01–0.34]	**0.43**	**[0.06–0.80]**	**0.53**	**[0.04–1.03]**	0.66	[−0.05–1.36]	**0.17**	**[0.00–0.33]**
Greece	9.07	[6.40–11.74]	**1.21**	**[0.08–2.34]**	**3.10**	**[0.86–5.34]**	**3.78**	**[0.76–6.80]**	**4.62**	**[0.30–8.94]**	**1.22**	**[0.17–2.26]**
Hungary	3.83	[2.28–5.38]	**0.51**	**[0.01–1.02]**	**1.34**	**[0.28–2.39]**	**1.64**	**[0.22–3.06]**	2.02	[−0.01–4.05]	**0.52**	**[0.04–0.99]**
Ireland	4.84	[2.99–6.69]	**0.65**	**[0.02–1.28]**	**1.68**	**[0.38–2.98]**	**2.06**	**[0.31–3.81]**	**2.53**	**[0.02–5.04]**	**0.65**	**[0.06–1.24]**
Italy	1.39	[0.43–2.34]	0.19	[−0.03–0.40]	**0.49**	**[0.01–0.97]**	0.60	[-0.03–1.23]	0.74	[−0.14–1.62]	0.19	[−0.02–0.39]
Latvia	3.69	[1.92–5.45]	0.50	[−0.01–1.00]	**1.29**	**[0.22–2.36]**	**1.58**	**[0.15–3.01]**	1.94	[−0.08–3.97]	**0.50**	**[0.02–0.97]**
Lithuania	2.26	[1.00–3.51]	0.30	[−0.02–0.63]	**0.79**	**[0.09–1.50]**	**0.97**	**[0.04–1.91]**	1.20	[−0.12–2.52]	**0.31**	**[0.00–0.61]**
Luxembourg	2.34	[1.20–3.48]	0.32	[−0.01–0.64]	**0.82**	**[0.13–1.51]**	**1.01**	**[0.08–1.93]**	1.25	[−0.07–2.56]	**0.32**	**[0.01–0.62]**
Malta	5.46	[3.66–7.25]	**0.73**	**[0.04–1.43]**	**1.89**	**[0.48–3.30]**	**2.32**	**[0.41–4.23]**	**2.85**	**[0.10–5.59]**	**0.74**	**[0.09–1.38]**
Montenegro	16.98	[13.70–20.26]	**2.23**	**[0.23–4.23]**	**5.63**	**[1.87–9.40]**	**6.82**	**[1.75–11.89]**	**8.26**	**[1.04–15.47]**	**2.25**	**[0.41–4.09]**
Netherlands	2.20	[1.08–3.31]	0.30	[−0.01–0.60]	**0.77**	**[0.11–1.43]**	**0.95**	**[0.06–1.83]**	1.17	[−0.08–2.42]	**0.30**	**[0.01–0.59]**
Norway	2.82	[1.72–3.92]	**0.38**	**[0.01–0.75]**	**0.99**	**[0.21–1.76]**	**1.21**	**[0.17–2.26]**	1.50	[−0.01–3.00]	**0.38**	**[0.04–0.73]**
Poland	5.82	[4.13–7.50]	**0.78**	**[0.05–1.51]**	**2.01**	**[0.55–3.48]**	**2.46**	**[0.47–4.46]**	**3.03**	**[0.15–5.90]**	**0.78**	**[0.11–1.46]**
Portugal	2.97	[1.36–4.58]	0.40	[−0.03–0.82]	**1.04**	**[0.13–1.95]**	**1.27**	**[0.07–2.48]**	1.57	[−0.13–3.27]	**0.40**	**[0.00–0.80]**
Romania	5.50	[3.87–7.12]	**0.74**	**[0.05–1.43]**	**1.91**	**[0.51–3.30]**	**2.33**	**[0.44–4.22]**	**2.87**	**[0.13–5.60]**	**0.74**	**[0.10–1.38]**
Serbia	10.60	[7.66–13.54]	**1.41**	**[0.10–2.71]**	**3.60**	**[1.04–6.16]**	**4.39**	**[0.93–7.84]**	**5.35**	**[0.41–10.29]**	**1.42**	**[0.21–2.62]**
Slovakia	4.15	[2.47–5.84]	**0.56**	**[0.01–1.11]**	**1.45**	**[0.31–2.59]**	**1.77**	**[0.24–3.31]**	2.18	[−0.01–4.38]	**0.56**	**[0.05–1.07]**
Slovenia	4.48	[3.12–5.84]	**0.60**	**[0.03–1.17]**	**1.56**	**[0.41–2.71]**	**1.91**	**[0.35–3.48]**	**2.35**	**[0.09–4.61]**	**0.61**	**[0.08–1.13]**
Spain	4.31	[3.26–5.36]	**0.58**	**[0.04–1.11]**	**1.50**	**[0.43–2.57]**	**1.84**	**[0.37–3.31]**	**2.26**	**[0.13–4.40]**	**0.58**	**[0.09–1.07]**
Sweden	2.76	[1.60–3.92]	**0.37**	**[0.00–0.74]**	**0.97**	**[0.19–1.74]**	**1.19**	**[0.14–2.23]**	1.46	[−0.03–2.96]	**0.37**	**[0.03–0.72]**
Switzerland	1.02	[0.36–1.69]	0.14	[−0.02–0.30]	**0.36**	**[0.02–0.71]**	0.44	[−0.01–0.90]	0.55	[−0.09–1.19]	0.14	[−0.01–0.29]
Turkey	25.46	[22.65–28.27]	**3.31**	**[0.42–6.19]**	**8.20**	**[3.00–13.39]**	**9.85**	**[2.91–16.80]**	**11.81**	**[2.01–21.61]**	**3.33**	**[0.68–5.97]**
UK	5.71	[4.31–7.12]	**0.77**	**[0.06–1.47]**	**1.98**	**[0.57–3.39]**	**2.42**	**[0.50–4.34]**	**2.98**	**[0.19–5.76]**	**0.77**	**[0.12–1.42]**
Total (35 countries)	5.13	[4.86–5.40]	**0.69**	**[0.08–1.30]**	**1.78**	**[0.59–2.97]**	**2.18**	**[0.53–3.83]**	**2.68**	**[0.25–5.11]**	**0.69**	**[0.13–1.25]**
*p* value	***		***		***		***		***		***	
Total (28 EU countries)	3.52	[3.18–3.87]	**0.47**	**[0.05–0.90]**	**1.23**	**[0.40–2.07]**	**1.51**	**[0.35–2.67]**	**1.86**	**[0.15–3.57]**	**0.48**	**[0.09–0.86]**
*p* value	***		ns		**		***		ns		*	

Bold: AF significantly different from 0 at 5%. *p* value for the comparison between countries: *p < 0.05; **p < 0.01; *** *p* < 0.001; *ns* non-significant

*FYROM* Former Yugoslav Republic of Macedonia, *Pe* prevalence of exposure, *AF* attributable fraction

The prevalence of long working hours was 5% in Europe (35 countries) and there were differences in this prevalence between countries. The lowest prevalence of exposure was observed in Austria, Germany, Italy, and Switzerland (less than 2%) and the highest in Turkey (more than 25%). The fraction of CHD attributable to long working hours was 1% and significantly different from zero. There were differences in this AF between the 35 countries but not between the 28 EU countries. The AF for overall stroke was 2% and was significant. There were differences in this AF between countries. The AF for atrial fibrillation was 2% and significant. There were differences in this AF between countries. The AF for venous thromboembolism was significant (3%), and there were differences between the 35 European countries, but not between the 28 EU countries. The AF for depression was 1% and significant. Differences in the AFs were observed between countries.

### Results for workplace bullying (Table 6)

**Table 6:** Fractions of mental disorders attributable to workplace bullying in Europe

%	Exposure prevalence		Depression	
	Pe^1^	95% CI	AF^2^	95% CI
Albania	0.28	[− 0.27–0.84]	0.52	[− 0.53–1.57]
Austria	6.02	[4.36–7.67]	**9.91**	**[5.76–14.06]**
Belgium	8.26	[6.98–9.54]	**13.12**	**[8.50–17.75]**
Bulgaria	0.15	[− 0.06–0.37]	0.28	[− 0.13-0.70]
Croatia	2.83	[1.48–4.18]	**4.93**	**[2.07–7.79]**
Cyprus	2.36	[1.20–3.52]	**4.15**	**[1.67–6.62]**
Czech Rep	1.93	[0.70–3.16]	**3.41**	**[0.95–5.87]**
Denmark	3.94	[2.58–5.29]	**6.72**	**[3.53–9.92]**
Estonia	2.79	[1.47–4.12]	**4.87**	**[2.05–7.69]**
Finland	5.54	[3.79–7.30]	**9.21**	**[5.10–13.32]**
France	12.79	[10.85–14.72]	**18.91**	**[12.72–25.10]**
FYROM	4.96	[2.99v6.92]	**8.31**	**[4.14–12.48]**
Germany	5.12	[4.02–6.23]	**8.58**	**[5.18–11.97]**
Greece	2.50	[1.19–3.80]	**4.37**	**[1.66–7.09]**
Hungary	1.05	[− 0.06–2.16]	1.88	[− 0.23–3.99]
Ireland	9.77	[7.00–12.55]	**15.13**	**[9.10–21.15]**
Italy	2.58	[1.46–3.70]	**4.52**	**[2.02–7.01]**
Latvia	4.92	[3.43–6.42]	**8.27**	**[4.60–11.93]**
Lithuania	4.64	[2.99–6.29]	**7.82**	**[4.08–11.55]**
Luxembourg	9.93	[7.73–12.13]	**15.35**	**[9.70–20.99]**
Malta	6.05	[4.29–7.82]	**9.96**	**[5.70–14.23]**
Montenegro	3.46	[1.96–4.95]	**5.95**	**[2.73–9.17]**
Netherlands	7.92	[5.88–9.96]	**12.64**	**[7.62–17.66]**
Norway	5.01	[3.53–6.48]	**8.40**	**[4.73–12.07]**
Poland	1.16	[0.41–1.90]	**2.08**	**[0.54–3.62]**
Portugal	0.80	[0.12–1.48]	**1.45**	**[0.11–2.80]**
Romania	4.12	[2.36–5.88]	**7.01**	**[3.29–10.73]**
Serbia	5.19	[3.44–6.95]	**8.68**	**[4.67–12.68]**
Slovakia	1.96	[0.78–3.14]	**3.47**	**[1.07–5.86]**
Slovenia	6.08	[4.65–7.51]	**10.01**	**[6.02–14.00]**
Spain	3.65	[2.78–4.52]	**6.27**	**[3.65–8.89]**
Sweden	4.44	[3.04–5.85]	**7.52**	**[4.10–10.95]**
Switzerland	4.11	[2.62–5.60]	**6.99**	**[3.59–10.40]**
Turkey	2.26	[1.36–3.16]	**3.98**	**[1.88–6.08]**
UK	5.35	[4.05–6.64]	**8.92**	**[5.29–12.55]**
**Total (35 countries)**	4.70	[4.45–4.95]	**7.93**	**[5.16–10.70]**
**p-value**	***		***	
**Total (28 EU countries)**	5.30	[4.88–5.72]	**8.85**	**[5.75–11.95]**
**p-value**	***		***	

Bold: AF significantly different from 0 at 5% *p*-value for the comparison between countries: ***: *p* < 0.001

*FYROM* Former Yugoslav Republic of Macedonia

^1^PE: Prevalence of exposure

^2^AF: Attributable fraction

The prevalence of bullying was 5% in Europe (35 countries) and there were differences between countries. Albania, Bulgaria, and Portugal had the lowest prevalence of exposure (less than 1%) and France the highest prevalence (more than 10%). The fraction of depression attributable to bullying was 7% and was significant. Differences in the AFs were found between countries.

### Gender differences (Supplementary Tables S3, S4)

There were some significant differences in the prevalence of exposure between men and women. Women were more likely to be exposed to workplace bullying than men in the total sample of the 35 European countries, whereas men were more likely to be exposed to ERI and long working hours than women in the total sample as well as in the sub-sample of the 28 EU countries (Supplementary Table S3). The corresponding AFs were calculated for men and women separately if gender differences in exposure were observed. Almost all the fractions attributable to long working hours were found to be significantly higher for men than for women (Supplementary Table S4). However, no gender differences were found in the AFs for ERI and bullying.

### Overall attributable fractions (Supplementary Table S5)﻿

The overall fraction of CHD attributable to all studied psychosocial work exposures ranged between 5% and 11% for the 35 European countries and for the 28 EU countries. The overall fraction of depression ranged between 17 and 35% for the 35 European countries and beween 16 and 35% for the 28 EU countries. The minimum values were obtained from the highest individual AFs (job insecurity for CHD and job strain for depression), under the assumption of total dependence between exposures. The maximum values were calculated using Miettinen’s formula, under the assumption of independence between exposures. Tetrachoric correlation coefficients between exposures ranged between 0.05 and 0.61, suggesting some level of dependence between exposures. Consequently, the true overall AFs were between the minimum and maximum values.

## Discussion

### Summary of the results

The highest significant AF was the fraction of depression attributable to job strain (17%) in Europe. The AFs of depression were higher than those of cardiovascular diseases (for all exposures except long working hours). The AFs of cardiovascular diseases were found to be lower and ranged from 1 to 11%. Most of the AFs were significantly different from zero, except for the job strain–stroke pair. Differences in the AFs were observed between countries for all exposure–outcome pairs related to the outcome of depression and also to the exposure to long working hours. More differences were found between the 35 European countries than between the 28 EU countries. Gender differences in the AFs were observed for long working hours, these AFs being higher among men than among women for all outcomes. The overall fractions of depression and CHD attributable to all studied psychosocial work exposures ranged between 17 and 35% and between 5–11%, respectively.

### Comparison with the literature

The prevalences of exposure observed in the present study were roughly in agreement with previous estimates for job strain, ERI, job insecurity, long working hours, and bullying from European and national surveys (Burr et al. 2003; de Smet 2005; Lesuffleur et al. 2014; Niedhammer et al. 2014a), although the definition of exposure and study period were not always similar between surveys.

The main comparison regarding AFs that can be done was with the results of our previous publication (Niedhammer et al. 2014a), although the present study covered more countries than the previous one (35 versus 31). In agreement with our previous results, the highest significant AF was found for job strain and depression with a similar magnitude (18% in 2005 and 17% in 2015). The differences were, however, found to be significant between countries in 2015, whereas they were not significant in 2005. The results were also consistent for the fractions of CHD attributable to job strain (4% in both 2005 and 2015). For both years, the fraction was significantly different from zero but without differences between countries. The two AFs related to ERI produced in this study were lower than the estimates obtained in our previous publication (12% in 2005 versus 2% in 2015 for CHD and 9% in 2005 versus 6% in 2015 for depression). This was explained by the use of lower, but more reliable, estimates of RRs based on a higher number of studies. The fraction of depression attributable to job insecurity obtained in this study was higher than our previous estimate (5% in 2005 and 9% in 2015), due to the use of a more recent and higher RR estimate.

The comparison with the literature can also be made with some rare previous studies that provided estimates of AFs, but not always for Europe and European countries. The study by Kivimaki et al. (Kivimaki et al. 2012) showed an estimate of 3.4% (95% CI 1.5–5.4) for the fraction of CHD attributable to job strain calculated from the data of 13 occupational cohort studies in 7 European countries, in line with our results. The study by LaMontagne et al. (Lamontagne et al. 2008) provided estimates of 13.2% for males (95% CI 1.1–28.1) and 17.2% (95% CI 1.5–34.9) for females for the fraction of depression attributable to job strain in Australia, which is also in agreement with our findings. The study by Nurminen and Karjalainen (Nurminen and Karjalainen 2001) was related to mortality (thus not strictly comparable to our study) in Finland and reported estimates of 15% among men and 10% among women for deaths related to depressive episode attributable to job strain. Some other studies in Denmark (Hannerz et al. 2009; Tuchsen et al. 2004) assessed attributable fractions using industry or industrial sector as an indirect marker of occupational exposures, which is a very different approach from ours, and did not allow to provide information about specific exposures. Hannerz et al. (Hannerz et al. 2009) found that the excess fraction for depressive episodes was 21% among women and 32% among men (interpreted as the fraction attributable to a non-optimum work environment). Similarly, Tuchsen et al. (Tuchsen et al. 2004) estimated that the excess hospitalisation fraction for diseases in the nervous system was 7% among women and 12% among men, and for circulatory diseases 12% among women and 10% among men.

Gender differences were explored in our study and suggested that there were differences between genders in the prevalence of exposure to some psychosocial work factors. However, there were no differences reported in the literature in the RR estimates between men and women (which did not mean that there was none, as the study of subgroup differences may suffer from a lack of statistical power). As the differences in RRs between genders were not systematically tested in the literature, a firm conclusion about the absence of gender differences may be difficult to achieve. Consequently, we may assume that the main source of differences in AFs between men and women could be related more to differences in the prevalence of exposure than to differences in RRs. The fractions attributable to long working hours showed significant higher values for men than for women for all outcomes.

### Limitations and strengths of the study

The strengths of the study were the following: We used the data from the 2015 EWCS that is a large survey at European level covering both men and women in all countries. Weighted data were used to make the extrapolation possible at European and national levels. We were able to provide estimates for the prevalence of exposure that were based on the same questionnaire and the same definition for exposure, i.e. that were comparable across Europe and between European countries. We were thus able to provide up-to-date estimates of exposure prevalence for Europe and each country (Niedhammer et al. 2012). Five exposures were studied: job strain, ERI, job insecurity, long working hours, and workplace bullying, that constitute major psychosocial work hazards in developed countries. We explored various health outcomes related to cardiovascular diseases and mental disorders, with more precise definition than in our previous publication (Niedhammer et al. 2014a). Estimates of AFs were provided for these exposures and outcomes, and our study may be one of the first to present fraction estimates of stroke, atrial fibrillation, peripheral artery diseases, and venous thromboembolism attributable to psychosocial work factors. Furthermore, we provided overall AF estimates for all exposures together, something that has never been done before. We studied a higher number of countries in Europe than previously, 35 versus 31 (Niedhammer et al. 2014a). Comparison was done between countries for both exposure prevalence and AF. Differences in the prevalence of exposure were found between countries. Differences in AFs were also observed between countries, and most of these differences were observed between the 35 countries, whereas there was almost no difference between the 28 EU countries, suggesting a higher gap between EU countries and non-EU/acceding countries than between EU countries themselves. This was confirmed by significant higher prevalences of exposure to job strain, ERI, and long working hours, and by significant higher AFs, especially for long working hours in non-EU countries compared to EU countries (Supplementary Tables S6, S7). We studied gender differences in exposure prevalence, RR, and AF. We used RRs that were extracted from the literature, with similar adjustment for covariates. The retained literature reviews provided no evidence of differences in RRs according to gender and age groups, but only a part of these reviews and meta-analyses tested these differences formally, and there may be a lack of statistical power in doing so. In the same way, only some very rare reviews tested and found differences in RRs according to SES (Heikkila 2020; Li 2020) and between countries or continents (Kivimaki et al. 2012; Virtanen 2018). Consequently, to the best of our knowledge, subgroup differences in AFs would be more related to differences in exposure prevalence than in RRs.

There were, however, some limitations in our study. Although the data were recent (2015 EWCS), our results could not capture the changes induced by the new crisis related to the COVID-19 pandemic that may have impacted the work environment drastically. As the EWCS did not include validated questionnaires, our measures of exposure to job strain and ERI may be considered as proxies, and may be imprecise. Indeed, the number and content of the items were not strictly identical in our study in comparison with the recommended questionnaires. Furthermore, the definition of exposure to job strain and ERI may be considered arbitrary (median cut-off for job strain and ratio over 1 for ERI). The measurement of job insecurity, long working hours, and bullying was based on one item only. The measurement of long working hours used a cut-off of 55 h a week, in order to be consistent with the literature providing the RRs. A cut-off of 48 h a week (in agreement with the 2003/88/EC European Working Time Directive) would have been more pertinent for Europe. All in all, we may assume that using this cut-off, the AFs would have been similar (i.e. low), as the prevalence of exposure would have been higher and the RRs lower. Furthermore, as there were slight changes in the questionnaire of the EWCS survey from 2005 to 2015, some items were either removed or added, which led to some little changes in the measure of exposures between our two studies, the one published in 2014 (Niedhammer et al. 2014a) and the present one. These slight changes are likely to lead to a higher assessment quality in 2015 compared to 2005, especially for ERI for which a higher number of items was collected in 2015 compared to 2005. We studied five exposures only, because for these exposures, we had both exposure prevalence using the EWCS and pooled RRs from previous literature reviews. Consequently, we may have neglected other exposures, such as organizational injustice for example. The assessment of exposure may be slightly different between the data of the 2015 EWCS and the primary studies included in the literature reviews. Furthermore, these primary studies may themselves be heterogeneous in the assessment of exposure and outcome (Supplementary Appendix). The results may not be strictly comparable for a given outcome in our study, as the definition was not strictly equivalent between literature reviews. For example, depression was defined by clinical depression in one review (Madsen et al. 2017), and by depressive symptoms in the others (Ronnblad 2019; Rugulies et al. 2017; Theorell 2015; Virtanen et al. 2018). In addition, a reporting bias may be suspected in the exposure–outcome associations, and such a bias may be higher for depression than for cardiovascular diseases. We studied outcomes related to cardiovascular diseases and mental disorders only, as psychosocial work factors were found to be associated with these outcomes with a high level of evidence in the literature. The RRs used to calculate the AFs were those adjusted for gender, age, and SES, or the closest adjustment. Nevertheless, not all RRs were adjusted for SES, which may lead to potential overestimated RRs without this adjustment. The formula used to calculate AF may lead to biased estimates if adjusted RR is used instead of unadjusted RR. However, given the data available, it was the “best possible method” (Nurminen and Karjalainen 2001). The calculation of attributable fractions implies causality between exposure and outcome, a condition which may be difficult to fulfil, as the level of evidence varied according the studied exposure-outcome associations. Finally, we could not claim to estimate the total burden of diseases attributable to psychosocial work exposures, as we studied a limited number of exposures and outcomes.

## Conclusion

Our estimation of the burden of cardiovascular diseases and mental disorders attributable to psychosocial work factors may be more underestimated than overestimated. Indeed, only a limited number of exposures and outcomes were studied. The highest burden was found for depression which was expected. The AFs of depression were particularly high for job strain and to a lesser extent for job insecurity and workplace bullying. There were differences between countries in these AFs suggesting that some countries may have a concerning burden. Although the magnitude of AFs for cardiovascular diseases were lower than those of depression, these AFs were significant and warrant more attention especially for job strain and job insecurity with CHD, and for job strain with peripheral artery disease. Differences in the AFs between countries were found for long working hours, suggesting that national legislation regarding working time may have an impact on the burden of cardiovascular diseases. Our results also showed that overall AFs taking all studied exposures into account may be noticeable for CHD and even substantial for depression. More research on the combined effects of multiple exposures on health outcomes would be needed to refine our estimates of overall AFs. More exploration of subgroup differences in RRs and AFs would also be informative, especially regarding gender, age, SES, and countries. This study may be helpful to guide prevention policies and establish priorities at national and European levels.

### Supplementary Information

Below is the link to the electronic supplementary material.

Supplementary file1 (DOCX 45 KB)

## Data Availability

The dataset used and analysed during the current study are available from the corresponding author on reasonable request.

## Appendix

Summary list of the items used to measure psychosocial work factors (European Working Conditions Survey, 2015).

## Items related to the job strain model factors

### Psychological demands (5 items)

–Working at very high speed

–Working to tight deadlines

–Not having enough time to get the job done

–Pace of work dependent on the work done by colleagues

–Interrupting a task to take on an unforeseen task

### Decision latitude (11 items)

#### Skill discretion (3 items)

–Monotonous tasks

–Learning new things

–Being able to apply own ideas in work

#### Decision authority (8 items)

–Working hours entirely determined by yourself

–Being able to choose or change your order of tasks

–Being able to choose or change your methods of work

–Being able to choose or change your speed or rate of work

–Having a say in the choice of your work colleagues

–Being able to take a break when you wish

–Arranging to take an hour or two off during working hours to take care of personal or family matters is very easy

–Being able to influence decisions that are important for your work

## Items related to the effort-reward imbalance model factors

### Effort (6 items)

–Working at very high speed

–Working to tight deadlines

–Not having enough time to get the job done

–Interrupting a task to take on an unforeseen task

–People working under your supervision, for whom pay increases, bonuses or promotion depend directly on you

–Long working hours

### Reward (9 items)

#### Esteem (5 items)

–Colleagues help and support

–Manager helps and supports

–Being treated fairly at workplace

–Employees appreciated when they have done a good job

–Receiving the recognition I deserve for my work

#### Job promotion (3 items)

–Duties corresponding well with present skills

–Getting paid appropriately

–Job offering good prospects for career advancement

#### Job insecurity (1 item)

–Fear to lose job in the next 6 months
