# Correction: Update of the fractions of cardiovascular diseases and mental disorders attributable to psychosocial work factors in Europe

**DOI:** 10.1007/s00420-023-01964-x

**Published:** 2023-03-21

**Authors:** Isabelle Niedhammer, Hélène Sultan-Taïeb, Agnès Parent-Thirion, Jean-François Chastang

**Affiliations:** 1 INSERM, Univ Angers, Univ Rennes, EHESP, Irset (Institut de Recherche en Santé, Environnement Et Travail), UMR_S 1085, Epidemiology in Occupational Health and Ergonomics (ESTER) Team, Angers, France; 2 Human Resources Department, School of Management, Université du Québec À Montréal (UQAM), Montréal, QC Canada; 3 European Foundation for the Improvement of Living and Working Conditions (EUROFOUND), Dublin, Ireland

**Correction: International Archives of Occupational and Environmental Health (2022) 95:233–247** 10.1007/s00420-021-01737-4

We have discovered an error in the fractions of depression attributable to workplace bullying presented in Table 6 in our recently published paper (Niedhammer et al. 2022). The corrected attributable fractions are showed in Table 6 (corrected). The prevalences of exposure to workplace bullying are unchanged. This error does not change the text and the conclusions of our paper.

**Table 6:** **(corrected)** Fractions of mental disorders attributable to workplace bullying in Europe

%	Exposure prevalence		Depression	
	Pe^1^	95% CI	AF^2^	95% CI
Albania	0.28	[− 0.27–0.84]	0.52	[− 0.53–1.57]
Austria	6.02	[4.36–7.67]	**9.91**	**[5.76–14.06]**
Belgium	8.26	[6.98–9.54]	**13.12**	**[8.50–17.75]**
Bulgaria	0.15	[− 0.06–0.37]	0.28	[− 0.13–0.70]
Croatia	2.83	[1.48–4.18]	**4.93**	**[2.07–7.79]**
Cyprus	2.36	[1.20–3.52]	**4.15**	**[1.67–6.62]**
Czech Rep	1.93	[0.70–3.16]	**3.41**	**[0.95–5.87]**
Denmark	3.94	[2.58–5.29]	**6.72**	**[3.53–9.92]**
Estonia	2.79	[1.47–4.12]	**4.87**	**[2.05–7.69]**
Finland	5.54	[3.79–7.30]	**9.21**	**[5.10–13.32]**
France	12.79	[10.85–14.72]	**18.91**	**[12.72–25.10]**
FYROM	4.96	[2.99–6.92]	**8.31**	**[4.14–12.48]**
Germany	5.12	[4.02–6.23]	**8.58**	**[5.18–11.97]**
Greece	2.50	[1.19–3.80]	**4.37**	**[1.66–7.09]**
Hungary	1.05	[− 0.06–2.16]	1.88	[− 0.23–3.99]
Ireland	9.77	[7.00–12.55]	**15.13**	**[9.10–21.15]**
Italy	2.58	[1.46–3.70]	**4.52**	**[2.02–7.01]**
Latvia	4.92	[3.43–6.42]	**8.27**	**[4.60–11.93]**
Lithuania	4.64	[2.99–6.29]	**7.82**	**[4.08–11.55]**
Luxembourg	9.93	[7.73–12.13]	**15.35**	**[9.70–20.99]**
Malta	6.05	[4.29–7.82]	**9.96**	**[5.70–14.23]**
Montenegro	3.46	[1.96–4.95]	**5.95**	**[2.73–9.17]**
Netherlands	7.92	[5.88–9.96]	**12.64**	**[7.62–17.66]**
Norway	5.01	[3.53–6.48]	**8.40**	**[4.73–12.07]**
Poland	1.16	[0.41–1.90]	**2.08**	**[0.54–3.62]**
Portugal	0.80	[0.12–1.48]	**1.45**	**[0.11–2.80]**
Romania	4.12	[2.36–5.88]	**7.01**	**[3.29–10.73]**
Serbia	5.19	[3.44–6.95]	**8.68**	**[4.67–12.68]**
Slovakia	1.96	[0.78–3.14]	**3.47**	**[1.07–5.86]**
Slovenia	6.08	[4.65–7.51]	**10.01**	**[6.02–14.00]**
Spain	3.65	[2.78–4.52]	**6.27**	**[3.65–8.89]**
Sweden	4.44	[3.04–5.85]	**7.52**	**[4.10–10.95]**
Switzerland	4.11	[2.62–5.60]	**6.99**	**[3.59–10.40]**
Turkey	2.26	[1.36–3.16]	**3.98**	**[1.88–6.08]**
UK	5.35	[4.05–6.64]	**8.92**	**[5.29–12.55]**
Total (35 countries)	4.70	[4.45–4.95]	**7.93**	**[5.16–10.70]**
*p* value	***		***	
Total (28 EU countries)	5.30	[4.88–5.72]	**8.85**	**[5.75–11.95]**
*p* value	***		***	

Bold: AF significantly different from 0 at 5%

*p* value for the comparison between countries: ***p < 0.001

*FYROM* Former Yugoslav Republic of Macedonia

^1^*Pe* prevalence of exposure

^2^*AF* attributable fraction
