# Supplementary material for: Update of the fractions of cardiovascular diseases and mental disorders attributable to psychosocial work factors in Europe
Source: Int Arch Occup Environ Health. 2021 Jun 28;95(1):233–47. doi: 10.1007/s00420-021-01737-4 (PMC8237556; doi:10.1007/s00420-021-01737-4)
Supplement: Supplementary file 1 — Supplementary file1 (DOCX 45 KB) [file 420_2021_1737_MOESM1_ESM.docx]

**Supplementary appendix** Measure of exposure and outcome in the 15 retained literature reviews and meta-analyses

| **Review/meta-analysis** | **Exposure** | **Outcome** |
| --- | --- | --- |
| Descatha et al. 2020 | Long working hours ≥55 hours a week (reference group: 35-40 h/week) | Stroke (any assessment method including self-report) |
| Dragano et al. 2017 | ERI (original questionnaire or proxies) | CHD (any assessment method including self-report) |
| Fransson et al. 2015 | Job strain (original questionnaire or proxies) | Stroke (national hospital admission and death registries) |
| Heikkila et al. 2020 | Job strain (original questionnaire or proxies) | Peripheral artery disease (hospital record) |
| Kivimaki et al. 2012 | Job strain (original questionnaire or proxies) | CHD (national hospital admission and death registries) |
| Kivimaki et al. 2015a | Long working hours ≥55 hours a week (reference group: 35-40 h/week) | CHD (any assessment method including self-report) |
| Kivimaki et al. 2017 | Long working hours (≥55 hours a week being the most commonly used definition) | Atrial fibrillation (electronic health records, hospitalisation, death, drug reimbursement registries, electrocardiogram) |
| Kivimaki et al. 2018 | Long working hours ≥55 hours a week (reference group: 35-40 h/week) | Venous thromboembolism (electronic records for hospitalizations and deaths in national registers) |
| Li et al. 2020 | Long working hours ≥55 hours a week (reference group: 35-40 h/week) | Ischemic heart disease (any assessment method including self-report) |
| Madsen et al. 2017 | Job strain (original questionnaire or proxies) | Clinical depression (diagnostic interview or hospital records) |
| Ronnblad et al. 2019 | Job insecurity or precarious/temporary employment (any questionnaire) | Depressive symptoms (any assessment method) |
| Rugulies et al. 2017 | ERI (original questionnaire or proxies) | Depressive disorders (any assessment method) |
| Theorell et al. 2015 | Bullying (any questionnaire) | Depressive symptoms (any assessment method) |
| Virtanen et al. 2013 | Job insecurity (any questionnaire) | CHD (any assessment method) |
| Virtanen et al. 2018 | Long working hours (≥55 hours a week in most studies) | Depressive symptoms (any assessment method) |

**Supplementary Table S1** Sample size of employees according to country, 2015 EWCS

|  | Total N | | Men N | | Women N | |
| --- | --- | --- | --- | --- | --- | --- |
| Albania | 553 | | 224 | | 329 | |
| Austria | 863 | | 379 | | 484 | |
| Belgium | 2178 | | 1051 | | 1127 | |
| Bulgaria | 886 | | 379 | | 507 | |
| Croatia | 822 | | 393 | | 427 | |
| Cyprus | 820 | | 410 | | 410 | |
| Czech Republic | 841 | | 380 | | 460 | |
| Denmark | 939 | | 471 | | 468 | |
| Estonia | 898 | | 355 | | 542 | |
| Finland | 792 | | 354 | | 437 | |
| France | 1392 | | 644 | | 747 | |
| FYROM | 716 | | 386 | | 330 | |
| Germany | 1840 | | 914 | | 925 | |
| Greece | 638 | | 350 | | 288 | |
| Hungary | 851 | | 387 | | 464 | |
| Ireland | 828 | | 380 | | 447 | |
| Italy | 956 | | 450 | | 505 | |
| Latvia | 841 | | 335 | | 506 | |
| Lithuania | 862 | | 339 | | 523 | |
| Luxembourg | 896 | | 444 | | 452 | |
| Malta | 880 | | 487 | | 393 | |
| Montenegro | 680 | | 370 | | 310 | |
| Netherlands | 864 | | 423 | | 441 | |
| Norway | 945 | | 421 | | 524 | |
| Poland | 1003 | | 435 | | 568 | |
| Portugal | 724 | | 302 | | 422 | |
| Romania | 853 | | 424 | | 429 | |
| Serbia | 689 | 344 | | 345 | |  |
| Slovakia | 873 | | 360 | | 513 | |
| Slovenia | 1354 | | 624 | | 730 | |
| Spain | 2762 | | 1341 | | 1421 | |
| Sweden | 926 | | 449 | | 477 | |
| Switzerland | 875 | | 447 | | 428 | |
| Turkey | 1365 | | 955 | | 410 | |
| UK | 1366 | | 702 | | 664 | |
| Total | 35571 | | 17109 | | 18453 | |

FYROM: Former Yugoslav Republic of Macedonia

**Supplementary Table S2** Fractions of ischemic stroke and hemorrhagic stroke attributable to job strain in Europe

| % | Exposure prevalence | | Ischemic stroke | | Hemorrhagic stroke | |
| --- | --- | --- | --- | --- | --- | --- |
|  | Pe^1^ | 95% CI | AF^2^ | 95% CI | AF^2^ | 95% CI |
| Albania | 39.43 | [34.68-44.18] | 6.60 | [-0.12-13.33] | -1.68 | [-12.77-9.41] |
| Austria | 22.45 | [19.23-25.68] | 3.90 | [-0.18-7.98] | -0.87 | [-7.13-5.39] |
| Belgium | 20.16 | [18.26-22.07] | 3.52 | [-0.15-7.19] | -0.77 | [-6.38-4.83] |
| Bulgaria | 21.30 | [18.27-24.33] | 3.71 | [-0.17-7.60] | -0.82 | [-6.76-5.11] |
| Croatia | 35.20 | [31.38-39.01] | 5.95 | [-0.14-12.03] | -1.46 | [-11.33-8.40] |
| Cyprus | 43.15 | [39.28-47.02] | 7.17 | [-0.08-14.42] | -1.87 | [-14.03-10.29] |
| Czech Rep | 26.79 | [23.29-30.29] | 4.61 | [-0.17-9.39] | -1.06 | [-8.54-6.41] |
| Denmark | 19.19 | [16.41-21.97] | 3.36 | [-0.17-6.89] | -0.73 | [-6.07-4.61] |
| Estonia | 21.28 | [18.00-24.57] | 3.71 | [-0.18-7.60] | -0.82 | [-6.75-5.11] |
| Finland | 16.31 | [13.56-19.06] | 2.87 | [-0.17-5.91] | -0.61 | [-5.15-3.93] |
| France | 24.33 | [21.82-26.83] | 4.21 | [-0.16-8.58] | -0.95 | [-7.73-5.82] |
| FYROM | 29.44 | [25.65-33.23] | 5.04 | [-0.17-10.24] | -1.19 | [-9.42-7.04] |
| Germany | 22.01 | [19.81-24.22] | 3.83 | [-0.15-7.81] | -0.85 | [-6.98-5.27] |
| Greece | 46.95 | [42.51-51.39] | 7.74 | [-0.05-15.53] | -2.07 | [-15.35-11.20] |
| Hungary | 31.44 | [27.77-35.12] | 5.36 | [-0.15-10.87] | -1.28 | [-10.08-7.52] |
| Ireland | 25.92 | [22.30-29.55] | 4.47 | [-0.18-9.12] | -1.03 | [-8.26-6.21] |
| Italy | 22.78 | [19.79-25.77] | 3.96 | [-0.17-8.08] | -0.89 | [-7.23-5.46] |
| Latvia | 15.16 | [12.45-17.88] | 2.68 | [-0.17-5.52] | -0.57 | [-4.79-3.66] |
| Lithuania | 27.56 | [24.01-31.10] | 4.73 | [-0.17-9.64] | -1.10 | [-8.80-6.60] |
| Luxembourg | 21.23 | [18.21-24.25] | 3.70 | [-0.17-7.57] | -0.82 | [-6.73-5.10] |
| Malta | 15.46 | [12.67-18.26] | 2.73 | [-0.17-5.63] | -0.58 | [-4.88-3.73] |
| Montenegro | 33.29 | [29.04-37.54] | 5.65 | [-0.16-11.45] | -1.37 | [-10.70-7.96] |
| Netherlands | 15.29 | [12.42-18.16] | 2.70 | [-0.17-5.57] | -0.57 | [-4.83-3.69] |
| Norway | 13.12 | [10.91-15.34] | 2.33 | [-0.15-4.81] | -0.48 | [-4.13-3.17] |
| Poland | 24.36 | [21.43-27.29] | 4.22 | [-0.16-8.60] | -0.96 | [-7.75-5.84] |
| Portugal | 26.16 | [22.37-29.95] | 4.51 | [-0.18-9.20] | -1.04 | [-8.34-6.27] |
| Romania | 36.83 | [32.76-40.90] | 6.20 | [-0.13-12.53] | -1.54 | [-11.88-8.79] |
| Serbia | 28.29 | [24.17-32.41] | 4.85 | [-0.18-9.89] | -1.13 | [-9.04-6.78] |
| Slovakia | 29.40 | [25.58-33.21] | 5.03 | [-0.17-10.23] | -1.19 | [-9.40-7.03] |
| Slovenia | 24.23 | [21.54-26.92] | 4.19 | [-0.16-8.55] | -0.95 | [-7.70-5.80] |
| Spain | 33.68 | [31.57-35.80] | 5.71 | [-0.12-11.54] | -1.39 | [-10.81-8.03] |
| Sweden | 16.07 | [13.46-18.68] | 2.83 | [-0.16-5.83] | -0.60 | [-5.07-3.87] |
| Switzerland | 21.44 | [18.45-24.43] | 3.73 | [-0.17-7.64] | -0.83 | [-6.80-5.14] |
| Turkey | 35.96 | [32.93-38.99] | 6.07 | [-0.12-12.25] | -1.50 | [-11.58-8.58] |
| UK | 27.26 | [24.47-30.05] | 4.69 | [-0.15-9.53] | -1.09 | [-8.69-6.52] |
| **Total (35 countries)** | 25.92 | [25.16-26.68] | 4.47 | [-0.14-9.08] | -1.02 | [-8.24-6.19] |
| **p-value** | *** |  | ns |  | ns |  |
| **Total (28 EU countries)** | 25.16 | [24.35-25.97] | 4.35 | [-0.14-8.83] | -0.99 | [-7.99-6.01] |
| **p-value** | *** |  | ns |  | ns |  |

FYROM: Former Yugoslav Republic of Macedonia

^1^Pe: prevalence of exposure

^2^AF: attributable fraction

Bold: AF significantly different from 0 at 5%

^3^p-value for the comparison between countries: *:p<0.05; **:p<0.01; ***: p<0.001; ns: non-significant

**Supplementary Table S3** Gender differences in the prevalence of exposure

| 35 European countries | Men |  | Women |  | p-value |
| --- | --- | --- | --- | --- | --- |
| Prevalence of exposure (%) | Pe | 95% CI | Pe | 95% CI |  |
| **Job strain** | 26.27 | 25.20-27.34 | 25.54 | 24.47-26.60 | ns |
| **Effort-reward imbalance** | 11.40 | 10.63-12.17 | 8.78 | 8.12-9.45 | *** |
| **Job insecurity** | 15.98 | 15.12-16.84 | 15.47 | 14.61-16.34 | ns |
| **Long working hours** | 7.86 | 7.20-8.52 | 2.73 | 2.31-3.15 | *** |
| **Workplace bullying** | 4.58 | 4.06-5.11 | 5.47 | 4.92-6.02 | * |
| 28 EU countries | Men |  | Women |  | p-value |
| Prevalence of exposure (%) | Pe | 95% CI | Pe | 95% CI |  |
| **Job strain** | 25.32 | 24.15-26.48 | 25.00 | 23.88-26.12 | ns |
| **Effort-reward imbalance** | 10.84 | 10.01-11.67 | 8.54 | 7.85-9.23 | *** |
| **Job insecurity** | 15.80 | 14.86-16.74 | 15.62 | 14.69-16.56 | ns |
| **Long working hours** | 5.10 | 4.51-5.69 | 1.95 | 1.60-2.29 | *** |
| **Workplace bullying** | 4.89 | 4.29-5.48 | 5.72 | 5.12-6.32 | ns |

p-value for the Rao-Scott Chi-Square test for the differences between genders *:p<0.05; **:p<0.01; ***: p<0.001; ns: non-significant

**Supplementary Table S4** Gender differences in the attributable fractions

| 35 European countries | Men |  | Women |  | p-value |
| --- | --- | --- | --- | --- | --- |
| Attributable fractions (%) | AF | 95% CI | AF | 95% CI |  |
| **Effort-reward imbalance** |  |  |  |  |  |
| CHD | 2.23 | 0.37-4.09 | 1.73 | 0.28-3.18 | ns |
| Depression | 7.21 | 4.19-10.24 | 5.66 | 3.24-8.07 | ns |
| **Long working hours** |  |  |  |  |  |
| CHD | 1.05 | 0.12-1.98 | 0.37 | 0.04-0.70 | * |
| Overall stroke | 2.70 | 0.90-4.50 | 0.96 | 0.30-1.62 | ** |
| Atrial fibrillation | 3.30 | 0.82-5.77 | 1.17 | 0.26-2.09 | * |
| Venous thromboembolism | 4.04 | 0.42-7.66 | 1.45 | 0.10-2.80 | * |
| Depression | 1.06 | 0.20-1.91 | 0.37 | 0.07-0.67 | * |
| **Workplace bullying** |  |  |  |  |  |
| Depression | 7.75 | 4.95-10.56 | 9.10 | 5.89-12.32 | ns |
| 28 EU countries | Men |  | Women |  | p-value |
| Attributable fractions (%) | AF | 95% CI | AF | 95% CI |  |
| **Effort-reward imbalance** |  |  |  |  |  |
| CHD | 2.12 | 0.35-3.90 | 1.68 | 0.27-3.09 | ns |
| Depression | 6.88 | 3.98-9.79 | 5.51 | 3.15-7.87 | ns |
| **Long working hours** |  |  |  |  |  |
| CHD | 0.68 | 0.07-1.29 | 0.26 | 0.02-0.50 | ns |
| Overall stroke | 1.77 | 0.57-2.97 | 0.68 | 0.21-1.16 | * |
| Atrial fibrillation | 2.17 | 0.51-3.82 | 0.84 | 0.18-1.50 | * |
| Venous thromboembolism | 2.67 | 0.23-5.10 | 1.04 | 0.06-2.01 | ns |
| Depression | 0.69 | 0.13-1.25 | 0.26 | 0.04-0.48 | * |

p-value for gender differences (Wald test)

The fractions attributable to job strain and job insecurity (and bullying for the 28 EU countries) were not calculated according to gender, as no gender differences in the prevalence of these exposures were observed.

**Supplementary Table S5** Individual and overall attributable fractions

| **ATTRIBUTABLE FRACTION (%)** | **35 countries** |  | **28 EU countries** |  |
| --- | --- | --- | --- | --- |
|  | **CHD** | **Depression** | **CHD** | **Depression** |
|  | **AF** | **AF** | **AF** | **AF** |
| **Individual AF for each exposure** |  |  |  |  |
| Job strain | 4.30 | 16.66 | 4.18 | 16.25 |
| Effort-reward imbalance | 1.93 | 6.28 | 1.91 | 6.21 |
| Job insecurity | 5.03 | 9.19 | 4.79 | 8.76 |
| Long working hours | 0.69 | 0.69 | 0.47 | 0.48 |
| Workplace bullying | - | 7.35 | - | 8.22 |
| **Overall AF for all exposures** |  |  |  |  |
| Min^1^ | 5.03 | 16.66 | 4.79 | 16.25 |
| Max^2^ | 11.48 | 34.74 | 10.93 | 34.54 |

^1^ Min overall AF = the highest individual AF for each exposure

^2^ Max overall AF from formula (2) in the method section

**Supplementary Table S6** Comparison in the prevalence of exposure between non-EU and EU countries

| Prevalence of exposure (%) | Non-EU |  | EU |  | p-value |
| --- | --- | --- | --- | --- | --- |
|  | Pe | 95% CI | Pe | 95% CI |  |
| **Job strain** | 31.17 | 29.09-33.25 | 25.16 | 24.35-25.97 | *** |
| **Effort-reward imbalance** | 13.22 | 11.64-14.80 | 9.70 | 9.16-10.25 | *** |
| **Job insecurity** | 15.88 | 14.31-17.45 | 15.71 | 15.05-16.37 | ns |
| **Long working hours** | 18.23 | 16.34-20.12 | 3.52 | 3.18-3.87 | *** |
| **Workplace bullying** | 2.98 | 2.33-3.64 | 5.30 | 4.88-5.72 | *** |

p-value for the Rao-Scott Chi-Square test for the comparison between non-EU and EU countries *:p<0.05; **:p<0.01; ***: p<0.001; ns: non-significant

**Supplementary Table S7** Comparison in the attributable fractions between non-EU and EU countries

| Attributable fractions (%) | Non-EU |  | EU |  | p-value |
| --- | --- | --- | --- | --- | --- |
|  | AF | 95% CI | AF | 95% CI |  |
| **Job strain** |  |  |  |  |  |
| CHD | 5.12 | 1.46-8.79 | 4.18 | 1.18-7.19 | ns |
| Overall stroke | 2.70 | -2.01-7.42 | 2.20 | -1.64-6.05 | ns |
| Peripheral artery disease | 12.66 | 4.84-20.47 | 10.50 | 3.88-17.11 | ns |
| Depression | 19.35 | 12.63-26.08 | 16.25 | 10.44-22.06 | ** |
| **Effort-reward imbalance** |  |  |  |  |  |
| CHD | 2.58 | 0.42-4.73 | 1.91 | 0.31-3.50 | ns |
| Depression | 8.26 | 4.76-11.77 | 6.21 | 3.59-8.83 | ns |
| **Long working hours** |  |  |  |  |  |
| CHD | 2.39 | 0.29-4.50 | 0.47 | 0.05-0.90 | ns |
| Overall stroke | 6.02 | 2.12-9.92 | 1.23 | 0.40-2.07 | * |
| Atrial fibrillation | 7.29 | 2.02-12.56 | 1.51 | 0.35-2.67 | * |
| Venous thromboembolism | 8.80 | 1.27-16.34 | 1.86 | 0.15-3.57 | ns |
| Depression | 2.41 | 0.48-4.34 | 0.48 | 0.09-0.86 | * |
| **Workplace bullying** |  |  |  |  |  |
| Depression | 5.19 | 3.05-7.33 | 8.85 | 5.75-11.95 | ** |

p-value for the comparison between non-EU and EU countries (Wald test)
